# Coffee Consumption and Whole-Blood Gene Expression in the Norwegian Women and Cancer Post-Genome Cohort

**DOI:** 10.3390/nu10081047

**Published:** 2018-08-09

**Authors:** Runa B. Barnung, Therese H. Nøst, Stine M. Ulven, Guri Skeie, Karina S. Olsen

**Affiliations:** 1 Department of Community Medicine, Faculty of Health Sciences, University of Tromsø-The Arctic University of Norway, 9037 Tromsø, Norway; therese.h.nost@uit.no (T.H.N.); guri.skeie@uit.no (G.S.); karina.standahl.olsen@uit.no (K.S.O.); 2 Department of Nutrition, Institute for Basic Medical Sciences, University of Oslo, P.O. Box 1046 Blindern, 0317 Oslo, Norway; smulven@medisin.uio.no

**Keywords:** whole-blood, mRNA, transcriptomics, gene expression, coffee, the Norwegian Women and Cancer Cohort (NOWAC)

## Abstract

Norwegians are the second highest consumers of coffee in the world. Lately, several studies have suggested that beneficial health effects are associated with coffee consumption. By analyzing whole-blood derived, microarray based mRNA gene expression data from 958 cancer-free women from the Norwegian Women and Cancer Post-Genome Cohort, we assessed the potential associations between coffee consumption and gene expression profiles and elucidated functional interpretation. Of the 958 women included, 132 were considered low coffee consumers (<1 cup of coffee/day), 422 moderate coffee consumers (1–3 cups of coffee/day), and 404 were high coffee consumers (>3 cups of coffee/day). At a false discovery rate <0.05, 139 genes were differentially expressed between high and low consumers of coffee. A subgroup of 298 nonsmoking, low tea consumers was established to isolate the effects of coffee from smoking and potential caffeine containing tea consumption. In this subgroup, 297 genes were found to be differentially expressed between high and low coffee consumers. Results indicate differentially expressed genes between high and low consumers of coffee with functional interpretations pointing towards a possible influence on metabolic pathways and inflammation.

## 1. Introduction

Coffee is consumed worldwide, and consumption rates in Norway (9.7 kg per capita) are surpassed only by Finland (12.3 kg per capita) [1]. On average, Norwegian women consume 454 grams of brewed coffee per day [2].

There has been a growing interest in studying the associations between coffee consumption and health in the recent decades. Some studies have indicated that coffee is beneficial to health, and it has been linked with a decreased risk of Alzheimer’s, Parkinson, and type 2 diabetes [3,4,5,6,7]. Studies have also indicated that coffee has either has a neutral or a beneficial effect on the risk of cancer, specifically associations with a probable decreased risk of liver, and endometrial cancer [8]. 

Other studies have revealed detrimental health effects such as increased total cholesterol and triglycerides in blood, as well as certain negative pregnancy outcomes [9,10,11,12,13]

These diverse health effects may be attributed to different constituents of coffee, some of the most bioactive being caffeine, cafestol, kahweol, polyphenols, trigonellin, and polycyclic aromatic hydrocarbons [14,15]. 

Linking the different coffee constituents to health outcomes is challenging because of the individual variation in metabolism and physiological response to coffee. As an example, the metabolism of caffeine can vary up to 12-fold between individuals, mostly due to the variability of hepatic cytochrome p450 *(CYP)1A2* activity, which metabolizes over 95% of caffeine [16].

Genes associated with either coffee or caffeine intake have been identified in genome-wide association studies of single nucleotide polymorphism (SNPs). Some of the most well established SNPs are located in *CYP1A1* and *CYP1A2* (caffeine metabolism), and *AHR* (regulation of *CYP1A2*) [17,18]. SNPs in these genes were also confirmed as being associated with coffee consumption in a large meta-analysis of over 120,000 individuals together with SNPs in six other genes (*GCKR*, *ABCG2*, *MLXIPL*, *POR*, *BDNF*, and *EFCAB5*) [19]. Still, the knowledge from functional genomics studies using mRNA is limited, and especially gene expression studies in peripheral blood are scarce.

The health effects of coffee consumption can also be difficult to disentangle from other diet and lifestyle factors, as many of the constituents of coffee are also present in other dietary sources. For example, tea and certain soft drinks contain caffeine, while smoking can influence the same metabolic pathways as coffee.

The Norwegian Women and Cancer Cohort (NOWAC) started its questionnaire data collection in 1991, with the aim of being a national representative, population-based cohort study [20]. Collection of whole-blood samples viable for microarray gene expression started in 2003 [21].

By using dietary data and whole-blood derived, microarray based mRNA gene expression data from NOWAC, we assessed whether high versus low consumers of coffee had differentially expressed genes that could elucidate the possible relevant biological processes associated with coffee consumption.

## 2. Materials and Methods

### 2.1. Study Population

**Figure 1:**
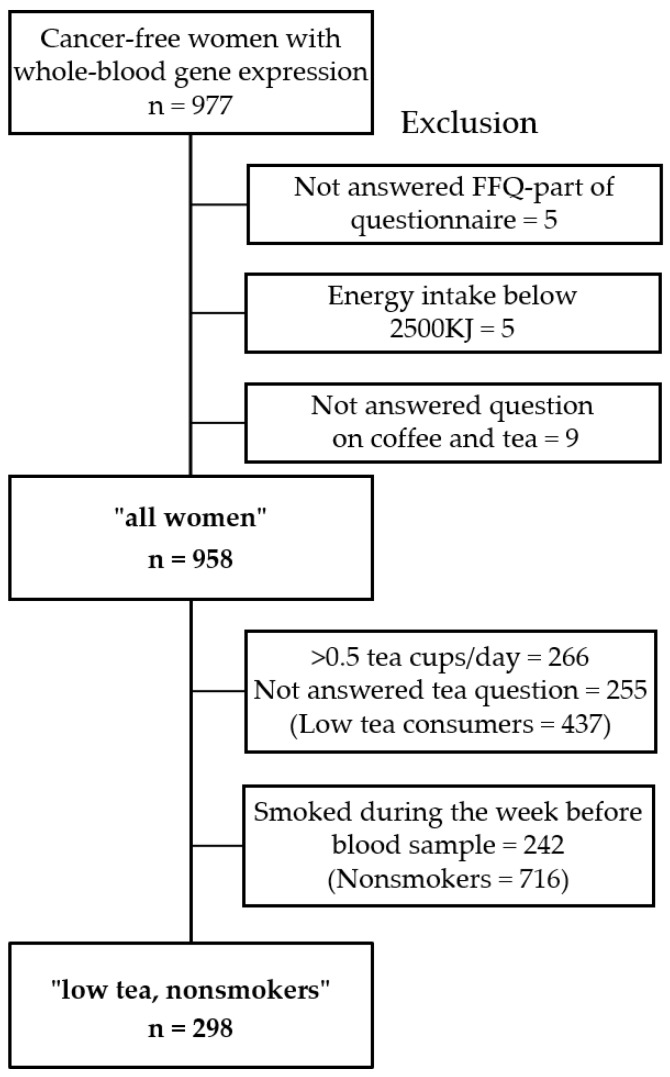
Flowchart showing the exclusion criteria in the study group and number of cancer-free women included in the study.

The NOWAC study consists of more than 170,000 women aged 30–70 years at recruitment. These women were randomly chosen from the Norwegian central person registry, and received an invitational letter and an eight-page lifestyle and food frequency questionnaire (FFQ). Approximately 50,000 of these women also later gave blood samples eligible for gene expression analysis (the Norwegian Women and Cancer Post-Genome Cohort), and answered a two-page questionnaire about current lifestyle at the time of blood sampling. Detailed information on NOWAC is available from Lund et al. [20], and on the NOWAC Post-Genome Cohort from Dumeaux et al. [21]. The present paper describes results from a subset of the NOWAC Post-Genome Cohort, where cancer-free women (*n* = 977) originally enrolled as controls in one prediagnostic- and one postdiagnostic breast cancer case-control study were included. These controls were randomly drawn, but matched by age and time of inclusion in the NOWAC cohort. Women who either (1) did not answer the food frequency part of the questionnaire; (2) or did not answer the questions regarding tea and coffee consumption or (3) consumed less than 2500 KJ were excluded. Further details about dietary assessment are given below. From the 977 women in total, 958 women were left in the group “all women” after exclusion criteria were applied (Figure 1). As smoking and tea consumption are highly confounding variables to coffee consumption, we performed a subgroup analysis of 298 nonsmokers who drank less than an average of half a cup of tea per day to isolate the effects of coffee from smoking and tea consumption.

The women gave written informed consent to donate blood samples for gene expression analysis. The NOWAC study was conducted in accordance with the Declaration of Helsinki, and approved by the Norwegian Data Inspectorate and the Regional Ethical Committee of North Norway (reference: REK NORD 2010/2075). Collection and storage of human biological material was approved by the REK in accordance with the Norwegian Biobank Act (reference: P REK NORD 141/2008 Biobanken kvinner og kreft ref. 200804332-3).

### 2.2. Determination of Gene Expression Levels

Non-fasting blood samples were collected using the PAXgene™ Blood RNA System (PreAnalytiX GmbH, CH–8634 Hombrechtikon, Switzerland), with buffers specially designed for the conservation of mRNA. The samples were mailed overnight to the Department of Community Medicine at the University of Tromsø-The Arctic University of Norway, and immediately frozen at −80 °C. The samples were sent to the Genomics Core Facility at the Norwegian University of Science and Technology, and processed according to the PAXgene Blood RNA Kit protocol. Total RNA was extracted and purified using the PAXgene Blood miRNA isolation Kit. RNA purity was assessed by NanoDrop ND 8000 spectrophotometer (ThermoFisher Scientific, Wilmington, DE, USA), and RNA integrity by Bioanalyzer capillary electrophoresis (Agilent Technologies, Palo Alto, CA, USA). Complementary RNA (cRNA) was prepared using the Illumina TotalPrepT-96 RNA Amplification Kit (Ambion Inc., Austin, TX, USA), and hybridized to Illumina HumanHT-12 Expression BeadChip microarrays (Illumina, Inc. San Diego, CA, USA). The raw microarray images were processed in Illumina GenomeStudio.

The preprocessing of the dataset was performed by the Norwegian Computing Center, and the methods are further described in Günter et al. [22]. In short, the preprocessing involved (1) removal of case-control pairs where either case or control was an outlier (determined by density plot, principal component analysis, or inspection of laboratory quality measures). (2) Background correction was performed using negative control probes (R package *limma*: Function nec), and finally (3) filtering out probes that either were reported to have poor quality in Illumina, were detectable in <1% of samples, or that were not annotated before mapping probes to genes. The dataset was then normalized on original scale by quantile normalization (R *lumi*: LumiN) and log2 transformed (R *lumi*: LumiT). The packages R *lumi*: nuID2RefSeqID and R *illuminaHumanv4.db* were used to annotate the preprocessed dataset. The final dataset included 7741 probes and 977 individuals.

### 2.3. Dietary Assessment and Descriptive Variables

The FFQ contains questions on quantity and frequency of the most commonly consumed food items. From these, grams per day (g/d) of the food items and total energy intake (kJ/d) were estimated. Standard portion sizes and weights were taken from the official Norwegian Weight and Measures for Foods [23], and intake of energy, alcohol and nutrients from the Norwegian Food Composition table [24]. The FFQ has been validated by test-retest reproducibility and by comparison with repeated 24-h dietary recalls [25,26]. The test-retest study concluded that the FFQ performed within the reported range for similar instruments, and the comparison with 24-h dietary recalls found that the FFQ gave a good ranking especially for foods consumed frequently. Coffee was found to have the best Spearman’s rank correlation coefficient (0.82) when the FFQ was compared to the 24-h dietary recalls [26].

Coffee consumption was self-reported based on the question: “How many cups of coffee do you normally drink of each brewing method?” with the different brewing methods being boiled, filtered, and instant. The frequency of consumption was divided into seven categories: Never/seldom, 1–6 cups per week, 1 cup per day, 2–3 cups per day, 4–5 cups per day, 6–7 cups per day, and 8+ cups per day. Interval midpoints of the frequencies were used to add the different brewing methods together. Average total coffee consumption was divided into the categories: Low (<1 cup/day), moderate (≥1–≤3 cups/day), and high (>3 cups/day). This categorization of coffee cups is similar to previously conducted studies on coffee consumption in the NOWAC cohort, but due to a lower sample population in the current study only one high consumption category was used [27,28].

A second version of the FFQ also included espresso (received by 205 of 977 women), only 9 of the 78 women who answered the question on espresso consumption replied something else than never/seldom. One cup of espresso was considered equal to one cup of coffee in the analyses.

One question on green tea and one question on black tea were combined for total tea consumption. For group characteristics, the variable g/d was used. However, the sum of the midpoints of the tea consumption frequency intervals was used for further establishing a subgroup “low tea, nonsmokers” that consisted of nonsmoking women who on average consumed less than half a cup of tea per day. This was done to isolate the effects of coffee from smoking and potential caffeine-containing tea consumption.

The women reported their physical activity level (both activity at home and at work) in the FFQ on a scale from 1 to 10, with 1 being very low and 10 being very high. Education was reported as years in school, including lower education. Both information on smoking status and BMI from self-reported height and weight were taken from the two-page questionnaire filled in at time of blood sampling. The smoking question asked if the women had smoked in the week prior to the blood sampling (yes/no).

### 2.4. Statistical Analysis

Potential confounders were investigated by comparing the categories of coffee consumption as described above using a Kruskal-Wallis test, robust ANOVAs, and a Chi-square test with *p* < 0.05 as the significance threshold; subsequent post hoc methods were then used to establish a significance between coffee consumption categories. Both Kruskal Wallis and robust ANOVA showed similar results, but since no variables were normally distributed except for “red and processed meat,” Kruskal-Wallis with Dunn’s post hoc rank sum test is presented in the tables. Based on these initial analyses, further analyses of differential gene expression between coffee consumption categories were performed on a subgroup of “low tea, nonsmoking” consumers (298 women), in addition to “all women.” In the “low tea, nonsmoking” group, the differences in age, education, and meat and dairy consumption found in the “all women” group were no longer significant, and were therefore not adjusted for.

All analyses were performed using R v3.4.0 [29] and packages from R and the Bioconductor project. The R package *limma* [30] was used to find differentially expressed genes (false discovery rate (FDR) < 0.05 was used) between the three categories of coffee consumption. The lists of differentially expressed genes from *limma* were then used in *clusterProfiler* [31] to perform over-representation analysis (R *clusterProfiler*: EnrichGO) and to compare the enriched functional categories of each gene cluster between “all women” and “low tea, nonsmokers” (R *clusterProfiler*: CompareCluster) for biological processes within Gene Ontology (GO) terms. To ensure balanced comparisons between the gene lists of each group, the top 100 genes in each list were used to compare the groups.

## 3. Results

### 3.1. Descriptors

The group “all women” consisted of 958 women with a median coffee consumption of 525 grams of brewed coffee per day. Of these 958 women, 132 (13.8%) had a low coffee consumption (<1 cup of coffee/day), 422 (44.1%) were moderate coffee consumers (≥1–≤3 cups of coffee/day), and 404 (42.2%) were high coffee consumers (>3 cups of coffee/day) (Table 1). Filtered coffee was reported as the brewing method by 783 women, followed by instant coffee (205 women), boiled coffee (121 women), and espresso (nine women), with some women consuming more than one type of brewing method.

There was a higher percentage of women who smoked in the week before the blood sample was taken in the high coffee consumption group (36.8%) compared to both the low (14.4%) and moderate coffee consumption groups (17.3%). The high coffee consumption group also had the lowest median tea intake (0 g/d) of the three groups. The moderate group had higher median tea consumption (135 g/d) than the high coffee consumers, but the low coffee consumption group had a substantially higher intake than both moderate and high coffee consumers with a median of 405 g/d.

Further, a low education level was more frequent in the high coffee consumption group than in the two other groups. There was a higher median intake of dairy products in the high (179 g/d) and moderate (175 g/d) coffee consumption groups compared to the low consumption group (128 g/d). Median consumption of red and processed meat was slightly higher in the high coffee consumption group (93 g/d), compared to the moderate (86 g/d) and low (86 g/d) consumption groups. However, for red and processed meat, the actual difference in grams was small, and this is therefore unlikely to be of clinical relevance.

Table 2 describes the characteristics of the subgroup of women who did not smoke in the week before blood sample donation, and that drank less than 1–6 cups of tea per week (average of half a cup per day). This “low tea, nonsmoking” group consisted of 298 women with a median coffee consumption of 630 grams brewed coffee per day, of which 25 (8.4%) had a low coffee consumption, 139 (46.6%) were moderate coffee consumers, and 134 (45.0%) were in the high coffee consumption category.

In the “low tea, nonsmoking” group there was a difference in median energy intake among the coffee consumption categories, with a borderline significant difference (*p* = 0.054) between the high (7188 kJ/day) and low consumption group (6450 kJ/day), and a significant difference between the moderate (6625 kJ/day) and high group (*p* = 0.034).

### 3.2. Differential Gene Expression

**Figure 2:**
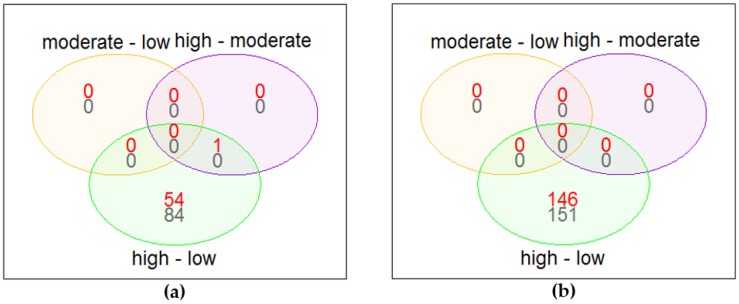
(**a**) Significantly up-(red) and down-(grey) regulated genes between coffee consumption categories for “all women.” (**b**) Significantly up-(red) and down-(grey) regulated genes between coffee consumption categories for “low tea, nonsmokers.”

When comparing high versus low coffee consumers in “all women,” there were 139 significantly differentially expressed genes (FDR < 0.05) (Figure 2a, Table S1). The gene most differentially expressed (*LRRN3*) when comparing high versus low coffee consumers was also the only differentially expressed gene when comparing high versus moderate coffee consumption groups. When studying only those who did not smoke the week before blood sampling, 414 genes were significantly differentially expressed between high and low consumers (results not presented). In the group that consisted of the 298 women who neither smoked in the week before blood sampling nor drank more than an average of half a cup of tea per day (“low tea, nonsmoking”), 297 genes were significantly differentially expressed when comparing high versus low coffee consumers (Figure 2b, Table S2). Table 3 shows the top 20 significantly differentially expressed genes when comparing high versus low coffee consumers in the “low tea, nonsmoking” group. There were 36 genes in common between all the significantly differentially expressed genes in “all women” and “low tea, nonsmoking” groups, but there was only one gene in common between the top 50 genes for both groups.

### 3.3. Over-Representation Analysis

**Figure 3:**
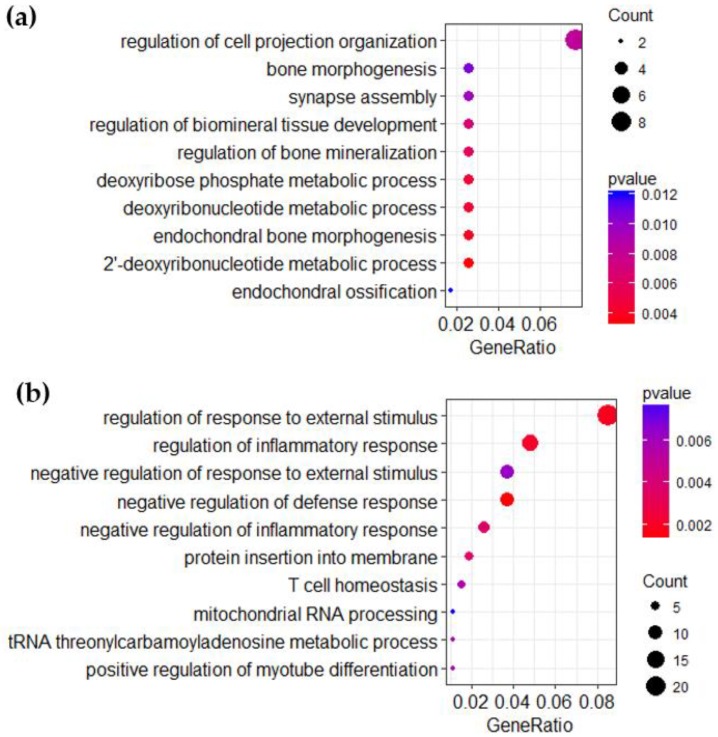
Over-representation analysis of Gene Ontology biological process categories. In the figure, the color of the dots indicates the *p*-value, the size of the dots indicates gene count, and the GeneRatio indicate the “number of genes in common between gene list and GO-category/number of genes in gene list.” (**a**) Over-representation analysis for “all women,” using the 139 significantly differentially expressed genes between high and low coffee consumers. (**b**) Over-representation analysis for “low tea, nonsmokers,” using the 297 significantly differentially expressed genes between high and low coffee consumers.

Over-representation analysis for the gene lists with significantly differentially expressed genes found no over-representation at FDR < 0.05. In the over-representation analysis for “all women” at *p*-value < 0.01 (*n* = 139 genes, Figure 3a), the top over-represented categories were involved in regulation and assembly of different tissues and cell constituents. In the “low tea, nonsmoking” group, processes related to immunological responses were indicated (*n* = 297 genes, Figure 3b). When separating the differentially expressed genes from the “low tea, nonsmoking” group into upregulated (146 genes) and downregulated (151 genes), the immunological responses were only apparent in the downregulated genes (Figure S1).

**Figure 4:**
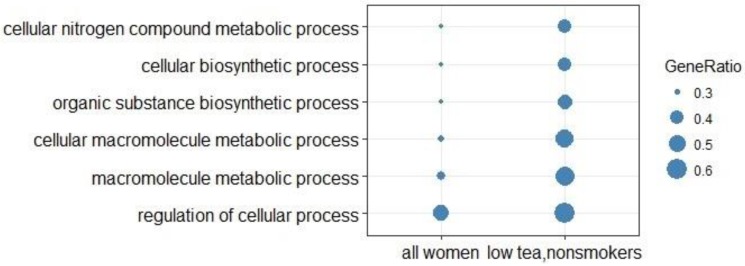
Group comparison of Gene Ontology biological process categories using a gene list of the top 100 significantly differentially expressed genes between high and low consumers in the “all women” group versus the “low tea, nonsmoking” group. GeneRatio indicates “number of genes in common between gene list and GO-category/number of genes in gene list.”

Genes related to metabolic processes were indicated in ontology categories in a group comparison of high and low coffee consumers between “all women” and “low tea, nonsmokers” when using the top 100 significantly differentially expressed genes for both groups (Figure 4).

## 4. Discussion

In this study of Norwegian women, 139 differentially expressed genes were found in whole-blood between self-reported high and low coffee consumers. Subgroup analyses with nonsmoking, low tea consumers yielded a separate set with 297 differentially expressed genes, but comparisons of the top 100 differentially expressed genes in both groups show similar tendencies towards gene ontologies involved in general metabolic processes. An over-representation analysis of GO biological process categories for the differentially expressed genes from the “low tea, nonsmoking” group pointed towards involvement in inflammation related processes. Both the “all women” and “low tea, nonsmoking” groups demonstrated modest fold changes, and the changes were both upregulation and downregulation of expression. This indicates effects from coffee consumption on whole-blood gene expression.

The median intakes of coffee consumption found in the current study were in accordance with the average consumption (560 g/d) among Norwegian women in the age group 50–59 [2]. Energy intake in the “low tea, nonsmoking” group was highest among high consumers of coffee. Few studies have investigated the influence of coffee consumption on energy intake. The studies that exist somewhat contradict our finding, with coffee consumption either having no effect on single meal energy intake or leading to a small daily decrease in energy intake [32].

Genes indicated from the gene expression profiles in this study have not previously been associated with coffee consumption. However, we were not able to distinguish the findings from coffee consumption in the full study group due to confounding from especially smoking. Smoking is strongly associated with coffee consumption, with smokers consuming more coffee than nonsmokers do, possibly due to an increased caffeine metabolism [33,34,35]. The two top differentially expressed genes (*LRRN3* and *PID1*) identified between current smokers and never smokers in a meta-analysis by Huan et al., [36] were the same two top differentially expressed genes between low and high consumers in the group “all women.” *LRRN3* was also the only gene differentially expressed between the moderate and high coffee consumers in the same group. The observation of *LRRN3* and *PID1* indicate a strong influence of smoking on the gene expression profiles for “all women.” However, *LRRN3* and *PID1* were not differentially expressed between high and low consumers of coffee in the “low tea, nonsmoking” group.

SNPs linked to several genes have previously been associated with coffee consumption [17,18,19], of these only *POR* was found to be significantly differentially expressed in the current study, and only in the group “low tea, nonsmokers.” *POR* encodes P450 oxidoreductase that transfers electrons to microsomal *CYP 450* enzymes, which are needed for the metabolism of caffeine [19].

Notably, some of the most prominent candidate genes (*CYP1A1*, *CYP1A2* and *AHR*) involved in caffeine metabolism were filtered out from our expression data due to low detection rates, and we were therefore not able to assess the association between these and coffee consumption in the NOWAC cohort. Still, the fact that these genes had low detection rates indicates low expression of these genes in whole-blood. *CYP1A2* is mainly expressed in the liver, and only low levels of *CYP1A1* can usually be found in lymphocytes [37,38]. The association found between *POR* and coffee consumption might indicate that the *CYP1* genes are affected in other ways than by transcriptional regulation in whole-blood. In general, genetic background must also be considered, especially sex and ethnicity can impact the expression of *CYP1A2* [39,40].

Among the top 20 differentially expressed genes from the “low tea, nonsmoking” group, there were especially five genes, *TXK*, *HLX*, *KDM6B*, *SPATA2L*, and *CDK5RAP1*, that are of interest for further research concerning coffee consumption and gene expression. *TXK* and *HLX* are involved in development of T-helper 1 cells, which are necessary for human immune defense [41,42]. *KDM6B*, also known as *JMJD3*, takes part in inflammatory responses by participating in differentiation of macrophages [43], while *SPATA2L* is involved in processes related to inflammatory signaling [44]. *CDK5RAP1* is a repressor of *CDK5*, which is a cyclin-dependent protein known to be involved in neurodegenerative diseases like Parkinson’s and Alzheimer’s [45,46]. However, among these five genes, only *TXK* was in the GO biological processes involving inflammatory responses found in the over-representation analysis.

Inflammatory response processes were indicated in the over-representation analysis on “low tea, nonsmokers.” It should be taken into consideration that monocytes and lymphocytes in whole-blood are immune cells, so an expression of immune-related processes should be expected, and is often found in studies concerning diet and gene expression [47,48]. Epidemiological studies have previously discovered that coffee consumption is associated with reduced risk of death attributed to inflammatory diseases, and that coffee consumption is negatively associated with inflammatory processes [49,50]. Another study found increased concentrations of inflammatory markers among both men and females that consumed >200 mL coffee per day compared to non-consumers [51]. Other indicated effects of coffee consumption have been. e.g., increased serum cholesterol [10], reduced risk of Parkinson’s disease [4,5,6], and reduced risk of type 2 diabetes [7], which are all health endpoints caused by inflammation. Thus, associations between high coffee consumption and inflammatory indicators in peripheral blood could indicate markers of related pathways. In the healthy Norwegian population over 60% of the antioxidant intake is estimated to originate from coffee [52]. The increased intake of antioxidants among coffee consumers is a plausible source for the positive influence of coffee on inflammatory processes. Negative influences on inflammatory processes might be related to cafestol and kahweol, two coffee lipids mainly found in unfiltered coffee. In particular, cafestol is associated with increased serum cholesterol, which is a known underlying factor of atherosclerosis [53,54].

Five GO categories of different biosynthetic and metabolic processes were found to be the top categories in the comparison between genes identified for “all women” and the “low tea, nonsmoking” group. The “low tea, nonsmoking” group had a higher proportion of the top 100 differentially expressed genes involved in the metabolic processes than the group “all women”. The metabolic processes evident in this comparison indicate that at least certain genes found to be associated with coffee consumption are involved in the metabolism of constituents of coffee. However, when looking at the over-representation analyses, these metabolic processes were not evident.

Some strengths and limitations of this study should be considered. Gene expression profiles represent a snapshot of the mRNA transcripts available in the whole-blood at the time of blood sampling, while the FFQ represent long term dietary intake. The indicated effects are therefore likely impacted by the discrepancy between this reported long term intake and short term mRNA snapshot. The blood samples were not collected in a fasting state, and we have no data on time since coffee consumption. Caffeine has a half-life of approximately 5.5 h, but other coffee metabolites have a half-life below one hour [55]. Therefore, both in the high and low consumers there could be participants whose gene expression profiles underestimates their differential expression compared to their FFQ reported intake.

This study used a relatively large number of women compared to many other whole-blood nutrigenomic studies, the higher sample size mitigates some of the concerns of limited impact and reliability found in other studies [48].

The FFQs used in this study were comprehensive and contained most of the commonly consumed food and beverages in Norway. However, there was no question designed to capture caffeine-containing beverages other than tea and coffee, and this might lead to some residual confounding in our analyses. Coffee consumption and other dietary exposures were assessed based on self-reported data. Thus, some misclassification could have occurred in the dietary exposures, although it was likely non-differential. The participants reported cups of coffee, but was not given an estimate of an average cup size, which would have allowed more detailed assessment of consumption. However, coffee showed good validation with a Spearman’s rank correlation coefficient of 0.82 when the FFQ was compared to the 24-h dietary recalls where the women reported coffee consumption either in exact amount or based on cup sizes from a picture booklet [26]. Coffee differs in chemical constituents depending on variables such as bean type, roasting of bean, grinding of bean, and soaking time of coffee grinds. This information was not available from the FFQ. Taken together, we cannot rule out the possibility of coffee category misclassification, and for some women the classification might differ between reported coffee consumption and some of its constituents due to difference in cup size, brewing strength, and other factors. In this paper, we focus on coffee per se, rather than its constituents, as this is what people consume. No biomarker assessed in blood was used to affirm the estimates of coffee.

The gene expression data was not adjusted for age, education, or consumption of red and processed meat or dairy, even though high coffee consumers reported lower education level and a higher intake of red meat and dairy. Smoking and tea consumption are two known confounders for coffee consumption, and were also associated with coffee consumption in this study. For that reason, subgroup analyses targeting women with low tea consumption (<0.5 cup/day) and no smoking in the week before blood sampling were performed. Subsequently, the associations found between coffee consumption and dairy, red and processed meat, age, and education disappeared, indicating that smoking might be driving the differences observed in the full study group, and not coffee consumption per se.

Whole-blood samples were used in the NOWAC post-genome cohort since these are relatively non-invasive and practical for cohort studies. The PAX gene Blood RNA System made it possible to ship blood samples by mail overnight without having to freeze them first, while at the same time conserving the mRNA over time. Whole-blood has been considered as a surrogate biopsy material for other tissues, due to its transporting role where it both interacts with all tissues and organs and is exposed to bioactive molecules such as nutrients, metabolites, pollutants, and waste products [56]. This makes whole-blood a viable candidate for capturing gene expression profiles associated with dietary exposure [56]. The most transcriptionally active blood cells are the leukocytes, which are important in immune responses. The gene expression microarrays were performed on whole-blood samples lacking information regarding disease status and immune cell subtypes. Gene expression profiles vary depending on differences in cellular components of the whole-blood [57], and infections or autoimmune diseases can introduce differences in these cellular components. By quantification of the blood composition, genes specific to immune cells could have been better elucidated.

## 5. Conclusions

In this exploratory cross-sectional study, we show that coffee consumption is significantly associated to differentially expressed genes in whole-blood. To the best of our knowledge this is the first study using mRNA gene expression data to elucidate how coffee consumption influences gene expression in whole-blood. Our results indicate that the differentially expressed genes between high and low coffee consumers were associated with both metabolic and inflammatory processes. Some of the top genes found to be differentially expressed are especially interesting in relation to the effect on inflammatory processes associated with coffee consumption, and warrant further investigation. However, since this is an exploratory cross-sectional study based on self-reported coffee consumptions, the results presented herein must be interpreted with care.

## Figures and Tables

**Table 1:** Descriptive statistics of “all women,” low coffee consumers, moderate coffee consumers, and high coffee consumers.

	All Women (*n* = 958)	Low Coffee Consumers (*n* = 132, 13.8%)	Moderate Coffee Consumers (*n* = 422, 44.1%)	High Coffee Consumers (*n* = 404, 42.2%)
Age, years **^,a^	55 (48–61, 958)	53 (47–60, 132)	55 (48–61, 422)	54 (48–60, 404)
BMI	25.1 (20.2–33.4, 941)	25.0 (19.8–33.7, 130)	25.0 (20.3–32.8, 418)	25.3 (20.2–33.8, 393)
Education (years in school) **^,a,b,c^	12 (8–18, 916)	13 (8–19, 128)	12 (8–19, 398)	11 (8–18, 390)
Smoking week before blood sample **^,a,c^				
Yes % (n)	25.1% (240)	14.4% (19)	17.3% (73)	36.8% (148)
No % (n)	74.9% (716)	85.6% (113)	82.7% (349)	63.2% (254)
Physical activity level	6 (3–9, 894)	6 (3–9, 122)	6 (3–9, 394)	6 (2–8, 378)
Energy intake (kJ/d)	7106 (4274–10409, 958)	7052 (4087–10705, 132)	7057 (4504–10094, 422)	7172 (4050–10817, 404)
Alcohol consumption (g/d)	2 (0–12, 958)	2 (0–13, 132)	2 (0–12, 422)	2 (0–12, 404)
Dairy intake (g/d) **^,a,b^	169 (15–635, 958)	128 (6–614, 132)	175 (20–634, 422)	179 (16–641, 404)
Black and green tea consumption (g/d) **^,a,b,c^	135 (0–945, 703)	405 (0–1512, 99)	135 (0–675, 309)	0 (0–675, 295)
Red and processed meat intake (g/d) **^,c^	89 (15–176, 958)	86 (10–174, 132)	86 (18–161, 422)	93 (23–185, 404)

Values presented as median (5 and 95 percentiles, number of women (*n*)) or % (*n*). Low coffee consumption (<1 cup/day), moderate coffee consumption (≥1–≤3 cups/day), and high coffee consumption (>3 cups/day). Categorical variables analyzed with χ^2^, continuous variables with Kruskal Wallis test, **: *p* ≤ 0.01, a: *p* ≤ 0.05 between low and moderate coffee consumption categories, b: *p* ≤ 0.05 between low and high coffee consumption categories, c: *p* ≤ 0.05 between moderate and high coffee consumption categories.

**Table 2:** Descriptive statistics of “low tea, nonsmoking women,” low coffee consumers, moderate coffee consumers, and high coffee consumers.

	Low Tea, Nonsmoking Women (*n* = 298)	Low Coffee Consumers (*n* = 25, 8.4%)	Moderate Coffee Consumers (*n* = 139, 46.6%)	High Coffee Consumers (*n* = 134, 45.0%)
Age, years	55 (47–61, 298)	55 (47–61, 25)	55 (47–61, 139)	55 (47–61, 134)
BMI	25.7 (20.4–33.0, 293)	25.8 (19.5–34.4, 24)	25.4 (20.4–33.0, 138)	26.2 (20.7–32.7, 131)
Education (years in school)	12 (8–18, 288)	14 (8–19, 24)	12 (8–18, 133)	12 (8–18, 131)
Physical activity level	6 (3–9, 282)	7 (2–9, 24)	6 (3–9, 131)	6 (3–8, 127)
Energy intake (kJ/d) **^,^^b^^,^^c^	6911 (4251–10455, 298)	6450(3742–9486, 25)	6625 (4440–9458, 139)	7188 (4309–10995, 134)
Alcohol consumption (g/d)	2 (0–12, 298)	3(0–13, 25)	2 (0–13, 139)	2 (0–9, 134)
Dairy intake (g/d)	188 (24–617, 298)	110 (18–491, 25)	191 (24–618, 139)	199 (28–623, 134)
Red and processed meat intake (g/d)	90 (12–169, 298)	80 (2–170, 25)	87 (14–148, 139)	95 (14–186, 134)

Values presented as median (5 and 95 percentiles, number of women (n)) or % (n). Low coffee consumption (<1 cup/day), moderate coffee consumption (≥1–≤3 cups/day), and high coffee consumption (>3 cups/day). Continuous variables with Kruskal Wallis test **: *p* ≤ 0.01, b: *p* ≤ 0.05 between low and high coffee consumption categories; c: *p* ≤ 0.05 between moderate and high coffee consumption categories.

**Table 3:** Top 20 significantly differentially expressed genes (false discovery rate < 0.05) between high and low coffee consumers in the “low tea, nonsmoking” group.

Gene Symbol	Gene Name	Log Fold Change	Average Expression	T	*p*-Value
*TLE3*	Transducin like enhancer of split 3	−0.318	6.907	−4.682	0.000004
*HLX*	H2.0 like homeobox	−0.299	7.276	−4.577	0.000007
*DDX18*	DEAD-box helicase 18	0.282	8.330	4.536	0.000008
*YRDC*	YrdC N6-threonylcarbamoyltransferase domain containing	0.159	7.034	4.415	0.000014
*KDM6B*	Lysine demethylase 6B	−0.294	7.003	−4.401	0.000015
*CANT1*	Calcium activated nucleotidase 1	−0.264	8.524	−4.397	0.000015
*WDR61*	WD repeat domain 61	0.213	7.608	4.387	0.000016
*MTSS1*	MTSS1, I-BAR domain containing	0.260	7.383	4.375	0.000017
*MACF1*	Microtubule-actin crosslinking factor 1	0.248	7.815	4.336	0.000020
*PPP3CC*	Protein phosphatase 3 catalytic subunit gamma	0.241	7.482	4.284	0.000025
*FAM36A*	Cytochrome c oxidase assembly factor	0.190	6.781	4.274	0.000026
*TXK*	TXK tyrosine kinase	0.265	7.023	4.261	0.000027
*TFE3*	Transcription factor binding to IGHM enhancer 3	−0.198	7.043	−4.230	0.000031
*SPATA2L*	Spermatogenesis associated 2 like	−0.220	7.134	−4.204	0.000035
*DYSF*	Dysferlin	−0.529	9.697	−4.197	0.000036
*TTC13*	Tetratricopeptide repeat domain 13	0.219	7.541	4.193	0.000036
*LOC642684*	-	−0.136	6.339	−4.188	0.000037
*CDK5RAP1*	CDK5 regulatory subunit associated protein 1	0.166	7.422	4.178	0.000039
*LOC441124*	-	−0.261	7.219	−4.175	0.000039
*PHOSPHO1*	Phosphoethanolamine/phosphocholine phosphatase	−0.400	7.532	−4.169	0.000040

Log Fold change: Log2 fold change between high and low coffee consumption; Average expression: Average log2-expression level for that gene; *t*: Moderated *t*-statistic.

## Supplementary Materials

The following are available online at http://www.mdpi.com/2072-6643/10/8/1047/s1, Table S1: Significantly differentially expressed genes (FDR < 0.05) “all women,” Table S2: Significantly differentially expressed genes (FDR < 0.05) “low tea-nonsmoking” group. Figure S1: Over-representation analyses of Gene Ontology biological process categories for up and down regulated genes in the “low tea, nonsmoking” group.

## References

[1] International Coffee Council (2012). Trends in Coffee Consumption in Selected Importing Countries.

[2] The Norwegian Directorate of Health (2012). Norkost 3. En Landsomfattende Kostholdsundersøkelse Blant Menn og Kvinner i Norge i Alderen 18-70 år, 2010-11.

[3] Liu Q.P., Wu Y.F., Cheng H.Y., Xia T., Ding H., Wang H., Wang Z.M., Xu Y. (2016). Habitual coffee consumption and risk of cognitive decline/dementia: A systematic review and meta-analysis of prospective cohort studies. Nutrition.

[4] Qi H., Li S. (2014). Dose-response meta-analysis on coffee, tea and caffeine consumption with risk of parkinson’s disease. Geriatr. Gerontol. Int..

[5] Hernan M.A., Takkouche B., Caamano-Isorna F., Gestal-Otero J.J. (2002). A meta-analysis of coffee drinking, cigarette smoking, and the risk of parkinson’s disease. Ann. Neurol..

[6] Costa J., Lunet N., Santos C., Santos J., Vaz-Carneiro A. (2010). Caffeine exposure and the risk of parkinson’s disease: A systematic review and meta-analysis of observational studies. J. Alzheimers Dis..

[7] Ding M., Bhupathiraju S.N., Chen M., van Dam R.M., Hu F.B. (2014). Caffeinated and decaffeinated coffee consumption and risk of type 2 diabetes: A systematic review and a dose-response meta-analysis. Diabetes Care.

[8] World Cancer Research Fund (2018). American Institute for Cancer Research. Continous Update Project Expert Report 2018. Non-Alcoholic Drinks and the Risk of Cancer..

[9] Jee S.H., He J., Appel L.J., Whelton P.K., Suh I., Klag M.J. (2001). Coffee consumption and serum lipids: A meta-analysis of randomized controlled clinical trials. Am. J. Epidemiol..

[10] Cai L., Ma D., Zhang Y., Liu Z., Wang P. (2012). The effect of coffee consumption on serum lipids: A meta-analysis of randomized controlled trials. Eur. J. Clin. Nutr..

[11] Grosso L.M., Bracken M.B. (2005). Caffeine metabolism, genetics, and perinatal outcomes: A review of exposure assessment considerations during pregnancy. Ann. Epidemiol..

[12] Greenwood D.C., Thatcher N.J., Ye J., Garrard L., Keogh G., King L.G., Cade J.E. (2014). Caffeine intake during pregnancy and adverse birth outcomes: A systematic review and dose-response meta-analysis. Eur. J. Epidemiol..

[13] Wikoff D., Welsh B.T., Henderson R., Brorby G.P., Britt J., Myers E., Goldberger J., Lieberman H.R., O’Brien C., Peck J. (2017). Systematic review of the potential adverse effects of caffeine consumption in healthy adults, pregnant women, adolescents, and children. Food Chem. Toxicol..

[14] Ludwig I.A., Clifford M.N., Lean M.E., Ashihara H., Crozier A. (2014). Coffee: Biochemistry and potential impact on health. Food Funct..

[15] Cano-Marquina A., Tarin J.J., Cano A. (2013). The impact of coffee on health. Maturitas.

[16] Cornelis M.C. (2015). Toward systems epidemiology of coffee and health. Curr. Opin. Lipidol..

[17] Cornelis M.C., Monda K.L., Yu K., Paynter N., Azzato E.M., Bennett S.N. (2011). Genome-wide meta-analysis identifies regions on 7p21 (ahr) and 15q24 (cyp1a2) as determinants of habitual caffeine consumption. PLoS Genet..

[18] Sulem P., Gudbjartsson D.F., Geller F., Prokopenko I., Feenstra B., Aben K.K. (2011). Sequence variants at cyp1a1-cyp1a2 and ahr associate with coffee consumption. Hum. Mol. Genet..

[19] Cornelis M.C., Byrne E.M., Esko T., Nalls M.A., Ganna A., Paynter N., Monda K.L., Amin N., Fischer K., The Coffee and Caffeine Genetics Consortium (2015). Genome-wide meta-analysis identifies six novel loci associated with habitual coffee consumption. Mol. Psychiatry.

[20] Lund E., Dumeaux V., Braaten T., Hjartaker A., Engeset D., Skeie G., Kumle M. (2008). Cohort profile: The norwegian women and cancer study--nowac--kvinner og kreft. Int. J. Epidemiol..

[21] Dumeaux V., Borresen-Dale A.L., Frantzen J.O., Kumle M., Kristensen V.N., Lund E. (2008). Gene expression analyses in breast cancer epidemiology: The norwegian women and cancer postgenome cohort study. Breast Cancer Res..

[22] Günter C., Holden M., Holden L. (2014). Preprocessing of Gene-Expression Data Related to Breast Cancer Diagnosis: Samba/35/14.

[23] Blaker B., Aarsland M. (1989). Mål og vekt for Matvarer.

[24] Norwegian Food Safety Authority, The Norwegian Directorate of Health, University of Oslo Norwegian Food Composition Database. www.matvaretabellen.no.

[25] Parr C.L., Veierod M.B., Laake P., Lund E., Hjartaker A. (2006). Test-retest reproducibility of a food frequency questionnaire (ffq) and estimated effects on disease risk in the norwegian women and cancer study (nowac). Nutr. J..

[26] Hjartaker A., Andersen L.F., Lund E. (2007). Comparison of diet measures from a food-frequency questionnaire with measures from repeated 24-hour dietary recalls. The norwegian women and cancer study. Public Health Nutr..

[27] Lukic M., Licaj I., Lund E., Skeie G., Weiderpass E., Braaten T. (2016). Coffee consumption and the risk of cancer in the norwegian women and cancer (nowac) study. Eur. J. Epidemiol..

[28] Gavrilyuk O., Braaten T., Skeie G., Weiderpass E., Dumeaux V., Lund E. (2014). High coffee consumption and different brewing methods in relation to postmenopausal endometrial cancer risk in the norwegian women and cancer study: A population-based prospective study. BMC Womens Health.

[29] R Core Team (2017). R: A Language and Environment for Statistical Computing.

[30] Ritchie M.E., Phipson B., Wu D., Hu Y., Law C.W., Shi W., Smyth G.K. (2015). Limma powers differential expression analyses for rna-sequencing and microarray studies. Nucleic Acids Res..

[31] Yu G., Wang L.G., Han Y., He Q.Y. (2012). Clusterprofiler: An r package for comparing biological themes among gene clusters. OMICS.

[32] Schubert M.M., Irwin C., Seay R.F., Clarke H.E., Allegro D., Desbrow B. (2017). Caffeine, coffee, and appetite control: A review. Int. J. Food Sci. Nutr..

[33] Bjorngaard J.H., Nordestgaard A.T., Taylor A.E., Treur J.L., Gabrielsen M.E., Munafo M.R., Nordestgaard B.G., Asvold B.O., Romundstad P., Davey Smith G. (2017). Heavier smoking increases coffee consumption: Findings from a mendelian randomization analysis. Int. J. Epidemiol..

[34] Swanson J.A., Lee J.W., Hopp J.W. (1994). Caffeine and nicotine—A review of their joint use and possible interactive effects in tobacco withdrawal. Addict. Behav..

[35] Grela A., Kulza M., Piekoszewski W., Senczuk-Przybylowska M., Gomolka E., Florek E. (2013). The effects of tobacco smoke exposure on caffeine metabolism. Ital. J. Food Sci..

[36] Huan T., Joehanes R., Schurmann C., Schramm K., Pilling L.C., Peters M.J., Magi R., DeMeo D., O’Connor G.T., Ferrucci L. (2016). A whole-blood transcriptome meta-analysis identifies gene expression signatures of cigarette smoking. Hum. Mol. Genet..

[37] Yi B., Yang J.Y., Yang M. (2007). Past and future applications of cyp450-genetic polymorphisms for biomonitoring of environmental toxicants. J. Environ. Sci. Health C Environ. Carcinog. Ecotoxicol. Rev..

[38] Spatzenegger M., Horsmans Y., Verbeeck R.K. (2000). Cyp1a1 but not cyp1a2 proteins are expressed in human lymphocytes. Pharmacol. Toxicol..

[39] Denden S., Bouden B., Haj Khelil A., Ben Chibani J., Hamdaoui M.H. (2016). Gender and ethnicity modify the association between the cyp1a2 rs762551 polymorphism and habitual coffee intake: Evidence from a meta-analysis. Genet. Mol. Res..

[40] McGraw J., Waller D. (2012). Cytochrome p450 variations in different ethnic populations. Expert Opin. Drug Metab. Toxicol..

[41] Kashiwakura J., Suzuki N., Nagafuchi H., Takeno M., Takeba Y., Shimoyama Y., Sakane T. (1999). Txk, a nonreceptor tyrosine kinase of the tec family, is expressed in t helper type 1 cells and regulates interferon gamma production in human t lymphocytes. J. Exp. Med..

[42] Mullen A.C., Hutchins A.S., High F.A., Lee H.W., Sykes K.J., Chodosh L.A., Reiner S.L. (2002). Hlx is induced by and genetically interacts with t-bet to promote heritable t(h)1 gene induction. Nat. Immunol..

[43] De Santa F., Totaro M.G., Prosperini E., Notarbartolo S., Testa G., Natoli G. (2007). The histone h3 lysine-27 demethylase jmjd3 links inflammation to inhibition of polycomb-mediated gene silencing. Cell.

[44] Schlicher L., Brauns-Schubert P., Schubert F., Maurer U. (2017). Spata2: More than a missing link. Cell Death Differ..

[45] Dhavan R., Tsai L.H. (2001). A decade of cdk5. Nat. Rev. Mol. Cell Biol..

[46] Reiter V., Matschkal D.M.S., Wagner M., Globisch D., Kneuttinger A.C., Muller M., Carell T. (2012). The cdk5 repressor cdk5rap1 is a methylthiotransferase acting on nuclear and mitochondrial rna. Nucleic Acids Res..

[47] De Mello V.D.F., Kolehmanien M., Schwab U., Pulkkinen L., Uusitupa M. (2012). Gene expression of peripheral blood mononuclear cells as a tool in dietary intervention studies: What do we know so far?. Mol. Nutr. Food Res..

[48] Olsen K.S., Skeie G., Lund E. (2015). Whole-blood gene expression profiles in large-scale epidemiological studies: What do they tell?. Curr. Nutr. Rep..

[49] Andersen L.F., Jacobs D.R., Carlsen M.H., Blomhoff R. (2006). Consumption of coffee is associated with reduced risk of death attributed to inflammatory and cardiovascular diseases in the iowa women’s health study. Am. J. Clin. Nutr..

[50] Schulze M.B., Hoffmann K., Manson J.E., Willett W.C., Meigs J.B., Weikert C., Heidemann C., Colditz G.A., Hu F.B. (2005). Dietary pattern, inflammation, and incidence of type 2 diabetes in women. Am. J. Clin. Nutr..

[51] Zampelas A., Panagiotakos D.B., Pitsavos C., Chrysohoou C., Stefanadis C. (2004). Associations between coffee consumption and inflammatory markers in healthy persons: The attica study. Am. J. Clin. Nutr..

[52] Svilaas A., Sakhi A.K., Andersen L.F., Svilaas T., Strom E.C., Jacobs D.R.J., Ose L., Blomhoff R. (2004). Intakes of antioxidants in coffee, wine, and vegetables are correlated with plasma carotenoids in humans. J. Nutr..

[53] Halvorsen B., Ranheim T., Nenseter M.S., Huggett A.C., Drevon C.A. (1998). Effect of a coffee lipid (cafestol) on cholesterol metabolism in human skin fibroblasts. J. Lipid Res..

[54] Hurtubise J., McLellan K., Durr K., Onasanya O., Nwabuko D., Ndisang J.F. (2016). The different facets of dyslipidemia and hypertension in atherosclerosis. Curr. Atheroscler Rep..

[55] Lang R., Dieminger N., Beusch A., Lee Y.M., Dunkel A., Suess B., Skurk T., Wahl A., Hauner H., Hofmann T. (2013). Bioappearance and pharmacokinetics of bioactives upon coffee consumption. Anal. Bioanal. Chem..

[56] Mohr S., Liew C.C. (2007). The peripheral-blood transcriptome: New insights into disease and risk assessment. Trends Mol. Med..

[57] Whitney A.R., Diehn M., Popper S.J., Alizadeh A.A., Boldrick J.C., Relman D.A., Brown P.O. (2003). Individuality and variation in gene expression patterns in human blood. Proc. Natl. Acad. Sci. USA.

